# The impact of pandemics on labour organization: insights from an Italian company archive during the Spanish Flu

**DOI:** 10.1007/s12076-023-00335-x

**Published:** 2023-03-17

**Authors:** Enrico Berbenni, Stefano Colombo

**Affiliations:** 1 Department of Modern and Contemporary History, Università Cattolica del Sacro Cuore, 20123 Milano, Italy; 2 Department of Economics and Finance, Università Cattolica del Sacro Cuore, 20123 Milano, Italy

**Keywords:** Pandemic, Spanish flu, Labour, Resilience, Italy, **s**: I10, N30

## Abstract

In this paper, we discuss the classical modelling approach of pandemics as a negative labour shock. We perform an archival analysis of one of the largest Italian banks (Credito Italiano) during the First World War – Spanish Flu period (1914–1920). In particular, we scrutinise the circulars that the central management of the bank sent out to the local branches, with the aim to assess whether the Spanish Flu has been perceived by contemporaries as an event seriously affecting personnel management. Though restricted to a single case-study, archival evidence does not support the existence of a remarkable negative labour supply shock affecting personnel management because of the Spanish Flu pandemic. Other war-related events probably increased the system’s resilience.

## Introduction

The current coronavirus pandemic has generated great interest toward previous pandemic episodes. Indeed, knowing the impact of past pandemics might provide useful insights to elaborate the most appropriate measures to face the current crisis. Spell and Bezrukova (2022), for instance, argue that histories of how past crises were managed can help us anticipate how today’s public health challenges will permanently change the workplace. However, the historical approach is often problematic for economists. Indeed, the most of pandemic episodes – with the important exception of the Spanish Flu – are so far in the time that having reliable data is almost impossible.^1^

Therefore, economists usually approach pandemics from an unhistorical perspective. Indeed, pandemics are mainly considered in the context of very abstract neoclassic macroeconomic models. Within such a purely theoretical structure, pandemic is then modelled as a negative labour supply shock because of the huge burden of victims caused by the pandemic.^2^ For instance, Boucekkine et al. (2008), in a highly influential paper, claim that “*an epidemiological shock is modelled by an initial shortfall in the size of population*” (p.6). More recently, Jordà et al. (2020) argue that the main consequence of a pandemic is given by “*the disproportionate effects on the labor force relative to land*” (p.8). Garret (2009) argues that “*the general conceptual foundation* […] *is that* [a pandemic] *results* […] *in a significant negative shock to manufacturing labour supply”* (p.712), while the European Commission (2006) claims that “*a permanent negative shock to the population level* […] *is the fundamental supply shock associated with the pandemic*” (p.8).

Assessing a pandemic as a labour supply negative shock allows deriving quite clear-cut implications. In particular, Boucekkine et al. (2008) argue that, by suddenly reducing the labour supply, a pandemic causes, in the very short-term, an increase of the per-capita income. However, after this income increase, if the fertility rate is not affected by the pandemic, the economy then goes back to the initial equilibrium, thus experiencing negative growth rates (i.e. a recession).^3^ The negative impact on the economy arising from the labour supply negative shock is found also in the so-called SIR (“susceptible, infected, and recovered” individuals) models, where the reduction of the labour supply is not caused only by the burden of deaths, but also by the micro-founded decisions of individuals to abstain or reduce their participation in labour activities in order to reduce the number of contacts and hence the probability of being infected (see for instance Eichenbaum et al., 2020).^4^

The Spanish Flu episode represents a useful test to discuss the modelling approach of pandemics: in the light of the historical evidence provided by the Spanish Flu, could a pandemic be simply interpreted as a negative labour shock? This approach has been questioned for example by Berbenni and Colombo (2021), where, by using a descriptive analysis of several macroeconomic variables in the post-pandemic period, it is shown that the impact of the Spanish Flu in Italy has been quite different from what predicted by theoretical models. A similar conclusion is reached also by Karlsson et al. (2014), which, by looking to Swedish data in the post Spanish Flu period, find no evidence of real wage variations, in contrast to the predictions of theoretical models that suggest an increase of the real wage because of the scarcity of labour.

In this paper, our focus is different from both Berbenni and Colombo (2021) and Karlsson et al. (2014). Indeed, we perform an *archival analysis* of one of the largest Italian banks (Credito Italiano) during the First World War – Spanish Flu period (1914–1920). The aim is to investigate whether the Spanish Flu has had relevant implications for the organization of labour. As long as the pandemic represents a significant and negative labour supply shock, one might expect relevant implications also for personnel management. At the best of our knowledge, this prediction has not been properly investigated yet in the literature. Hence, our archival inspection provides a first step to fill this gap.

In particular, we scrutinize the letters that the Central Management of the bank sent to the local branches, with the aim to assess whether the Spanish Flu has been perceived by the contemporaries as an event that was able to modify the labour organization. The instructions sent by Central Management to the branches on personnel management are reconstructed chronologically. The timing with which the circulars were sent allows even more communications to be traced each month. By focusing on the main topics addressed in the letters and on the provisions taken to deal with the emergence of workforce shortages, it is thus possible to reconstruct the evolution over time - month by month - of the problems associated with personnel management. Identifying how the phenomenon was perceived by contemporaries can help reducing – even if not resolving – the main challenges arising from the absence of sufficiently large and reliable quantitative data, i.e. causality and endogeneity problems.

With respect to data analysis, archival analysis has several advantages. The chronological succession of the letters allows distinguishing, for example, the real effects on the labour market of the different waves of the pandemic, which did not occur uniformly over the months. Besides, the circulars sent to the branches allow grasping the complexity of the factors and the events – not attributable to the pandemic alone – that influenced the personnel management of a large banking institution, whose branches were scattered throughout the country. Furthermore, as pointed out by Mariotti (2022), it might be difficult for “*econometrics and other quantitative exercises […] to unravel the complex and causal relationships at play. […] Historical events and their chains of cause-effect relations can provide explanations that go beyond their time to help understand subsequent events and outcomes*” (p.771).

The analysis, while suggesting a particular methodological approach, has the limitation of being restricted to a single case study – one firm/one sector/one country. Therefore, further evidence and international comparison are needed before generalizations can be drawn. However, our tentative conclusion is that the impact of the Spanish Flu on the organization of labour in Italy could have been less remarkable than expected for several reasons. First, despite the great number of victims, the deadliest wave of the Spanish Flu pandemic lasted only few months. Second, the simultaneous end of the Great War provoked the immediate demobilization of the army that overwhelmed the negative, if any, impact of the pandemic on the labour supply. Third, the Great War-induced change in the workforce composition (i.e. the massive participation of women in the job market) probably contributed to make the system more resilient in the face of exogenous shocks. Hence, archival evidence does not support the existence of a remarkable negative supply shock as a consequence of the Spanish Flu pandemic, at least in Italy, because of the existence of other concomitant events. In this sense, our archival analysis suggests that, in order to have the complete picture of the forces at work, any pandemic episode should be analysed by taking into account all the relevant historical aspects.

There is a bunch of papers that address the spatial dimension of the implications of the Spanish Flu while focusing on different aspects. Barro and Ursúa (2008) perform an econometric exercise to estimate the capacity of several historical events – namely, World War I, World War II, the Spanish Flu pandemic, the Great Depression, and post-World War II crisis (as the Latin America debt crisis) – of provoking GDP “disasters” (see Barro and Ursúa, 2008, p.1). It is found that, for several countries, the impact of World War I has been much greater than that of the influenza pandemic. This is true, for instance, for UK, France, and Germany, whereas for the US, which have been quite immune from the consequences of Great War, the Spanish Flu provoked a greater contraction of the GDP. Overall, Barro and Ursúa (2008) conclude that “*the most serious economic disaster in terms of incidence and severity of declines in […] GDP was World War II. This event was followed in terms of economic impact by World War I and the Great Depression of the early 1930s—which had similar overall consequences*” (p.15). Notably, the Spanish Flu is not mentioned in this ranking. Hence, the results in Barro and Ursúa (2008) are in line with our archival evidence (even if limited to the Italian case), where the Spanish Flu seems to be perceived by the contemporaries as less impacting by far than the World War I in terms of labour organization.

At the opposite, our conclusions are in contrast with the estimations reported by Barro et al. (2020). Indeed, Barro et al. (2020) – somehow questioning their previous conclusions in Barro and Ursúa (2008) – estimate the impact on GDP growth only of the World War I and the Spanish Flu, and conclude that “*the Great Influenza Pandemic is estimated to have reduced real per capita GDP by 6.2% in the typical country* [while] *World War I is estimated to have reduced real per capita GDP in the typical country by 8.4%*” (pp.11–12). Though restricted to the implications on labour organization in Italy, our archival evidence does not suggest a dramatic impact of the Spanish Flu, whereas it seems to confirm the importance of the Great War in terms of labour supply (and hence personnel management). Consequently, archival evidence points out to a much greater impact of the latter also in terms of GDP reduction.^5^

For a more direct comparison, our work could be related to Karlsson et al. (2014), which consider the impact of the Spanish Flu in Sweden. Notably, Sweden has not been directly involved in the World War I, so there are no confounding effects related to the war that might add to the implications of the pandemic.^6^ In particular, Karlsson et al. (2014) find that the Spanish Flu had a significant effect on the labour supply of females and minor, that have been asked to replace the adult males that deceased due to the influenza. Even if the econometric results in Karlsson et al. (2014) do not allow disentangling the implications of such a replacement in terms of work organization, they echo what happened in Italy as a consequence of the World War I, rather than the Spanish Flu. In other words, one might suspect that, in the absence of the war event, labour supply – and, consequently, work organization – would have been more responsive to the pandemic episode than it really was.^7^^,^^8^

The rest of the paper proceeds as follows. In Sect. 2 we reconstruct the epidemiological framework of the Spanish Flu in Italy. In Sect. 3 we provide the archival analyses. Section 4 discusses and concludes.

## The spanish flu in Italy

The study of the social and economic effects of the Spanish Flu in Italy encounters difficulties for various reasons. First of all, there is a problem of misreporting, which makes it practically impossible to be confident about data on the influenza’s contagiousness and mortality: Johnson and Mueller (2002) argue that limitations about the number of infections and deaths include “*nonregistration, missing records, misdiagnosis, and nonmedical certification, and may also vary greatly between locations*” (Johnson and Mueller 2002). Moreover, the flu was not one of the diseases for which compulsory reporting was required.

In this regard, it must also be remembered that during the war a general increase in the mortality rate took place in Italy, with the exacerbation of infectious diseases such as tuberculosis, malaria, cholera, and typhus. Worsened sanitation conditions and a weakened population due to food restrictions favoured the deterioration of the health framework.

To get an idea of how this latter upsets the trend of mortality among the civilian population, the best way is to resort to the calculations of the demographers who refer to the death surplus in the autumn-winter of 1918–1919 compared to the pre-war averages: estimates converge towards an impressive number of victims between over 300,000 and 600,000 (Tognotti 2015, p. 38–39, 172–173; Cosmacini, 2005, p. 362–363).

It is therefore quite surprising that, faced with such an extreme scenario, there has been a sort of removal of the pandemic from the memory and experience of contemporaries. The period in which the flu spread the most was not favourable for the news of the infection to receive the maximum diffusion. Military censorship rigorously avoided the spread of alarming news about the real health conditions of the country in a crucial phase such as the last months of the conflict. Furthermore, there was probably an almost instinctive reaction of defusing death and obscuring private mourning in the face of the tragedy of war (Tognotti 2015, p. 25–28). Still, the flu had devastating effects within just a few months, even if the estimates of real mortality on a global scale appear extremely controversial, ranging from 17 million to 100 million globally (Spreeuwenberg et al. 2018).

Despite the silence of the official press of the time, which minimized the real gravity of the situation, the sudden surge in daily mortality in some Italian cities in October 1918 is striking: in Naples, on 7 October, 256 deaths were recorded compared to a daily average of 45–50; in Rome, on October 15, 196 vs. 40–45; in Milan, on October 10, 171 vs. 38; in Turin, on October 15, 126 vs. 20–25 (Donelli and Di Carlo 2016, p. 238).

The infection rate – which also might play a fundamental part in creating difficulties for work organization – has been impressive. Taubenberger and Morens (2006) argue that “*an estimated one third of the world’s population (or ≈ 500 million persons) were infected and had clinically apparent illnesses during the 1918–1919 influenza pandemic*”, while in Italy the number of infected people fluctuated between five and six million individuals: taking into account that the Italian population was then about 35 million, the Spanish Flu hit one in seven Italians between the end of the 1918 summer and the 1919 winter (Mortara 1925, p. 380; Tognotti, 2015, p. 173–174).

The obscuration to which the Spanish Flu was subjected clashed with the real experience of ordinary people, which dramatically emerged in the letters sent – and rigorously censored by the authorities – from Italian citizens to relatives and friends residing abroad. Thus wrote a Sicilian woman in a letter to Buenos Aires:

*We are living in very sad times […] Hunger, plague, war. Throughout Italy there is a great pandemic called Spanish fever which also hit Monterosso; you cannot imagine how many young people die, if it lasts a long time anyone will not survive […]. We die like animals without the comfort of relatives and friends […] (*Tognotti, 2015, p. *148–149)*.

The Spanish Flu, like other pandemics, occurred in successive waves but – if compared for example with the current Covid-19 – the moment of greatest virulence was concentrated in a relatively short period, between September and November 1918. In fact, a first wave had occurred in the spring of that year but had gone overall almost unnoticed and with limited damage. The second wave, however, that of autumn, was by far the worst, with a surge in infections and deaths. Between the end of December and the beginning of 1919 there was a third *poussée* with less diffusion but with a fair number of fatal cases. Finally, at the beginning of 1920 there was a final resurgence of cases, coinciding with the epilogue of the pandemic.^9^

## Archival insights

The analysis of the impact on the labour market must necessarily start from the data illustrated in Sect. 2, but cannot be confined to them. The timing with which different events (the trinomial war, pandemic and post-war crisis) crossed each other has conditioned the way in which the influenza affected the organization of work.

Especially in the autumn of 1918, one could expect severe difficulties on the labour market, but due to the silence of contemporary official sources, sample surveys in company archives can confirm this hypothesis or not. This is a hitherto quite neglected research strand that could provide useful interpretative insights to complement quantitative analyses on the effects of the influenza on the growth rate of the economy (Carillo and Jappelli 2022). Furthermore, it has the advantage of escaping the censorship of time and thus provides a realistic representation of what was happening. Finally, by offering the view of the contemporaries about the impact of the Spanish Flu on labour organization, it helps alleviating estimation problems related to the scarcity of data.

Following this methodological approach, we then analyse the archive of a major Italian bank of the time, Credito Italiano, which is an exemplary case study in the service sector, also due to its widespread presence throughout Italy. Credito Italiano was a typical German-style universal bank, with strong connections with the industrial world and a branched territorial presence (Confalonieri 1994; Barucci 2021): in 1919 it had 59 branches in Italy, in addition to the London branch, and a total number of employees growing sharply from 2,286 to 1915 to 6,449 in 1920.

Specifically, we consider the documents titled “*Istruzioni alle filiali (Instructions to the Branches)*”, i.e. the circulars that the central management of the bank periodically sent to its branches throughout the country, in which operational indications were provided on the subject of personnel management.

The chronological analysis of the circulars issued between the end of 1914 (the outbreak of the war in Europe) and 1920 (the last wave of Spanish Flu) highlights some fundamental points.

First, the frequency of the instructions that the bank transmitted to the branches during the entire conflict illustrates the significant impact of the war on the organization of work (Ermacora 2017; Tomassini 2015; for a comparative framework see Horne, 2007). Already in May 1914 and then again in December of the same year (therefore even before Italy entered the war, May 1915), the central management highlighted the “*very considerable demand of personnel that is required in some of our offices*” and encouraged the branches to accept as many applications for employment as possible.^10^ The situation worsened in the following months due to the calls to arms, the effects of which began to be felt on the internal organization of the bank. The circulars stressed two consequences. The first is the deterioration of the quality of personnel. Indeed, it was recommended to

*examine with great breadth any application for a job that presents even some probability of a successful outcome, taking on as temporary employees those applicants who, despite lacking school qualifications, may turn out to be practically useful*.^11^

Selection criteria became looser, and the proportion of temporary staff presumably increased.

The second consequence was to increase female employment with an accountant’s diploma: the first recommendation to branches dates to August 1915, so it was a trend strictly connected with the war from the very beginning (Curli 1998).^12^ This need is expressed very clearly in a circular of June 1916:

*From the experience gained by other branches, we have been convinced of the convenience of hiring the greatest number of young ladies with the diploma of accountant or equivalent qualification in these moments, which, after a short internship, can advantageously substitute as a quality of work, if not as a quantity, male staff*.^13^

Another provision was the practice of addressing schools directly to encourage students, once they had completed their studies, to apply for employment at Credito Italiano. In June 1916, for example, the central management wrote to the branches as follows:

*In the present circumstances, the need to intensify the recruitment of personnel not subject to immediate future military obligations is more urgent than ever; we therefore ask you to arrange for your chief of staff to visit the managers of the aforementioned local schools, in order to have the graduates exempt from upcoming military calls to apply for employment at our institution*.^14^

The search for new staff in schools, even before young people had entered the job market, points out the existence of strong competition among companies to grab staff during wartime.

It is therefore noticeable that Credito Italiano – like presumably every other company – was forced by the war to take important decisions, dictated by the shortage of personnel caused by the repeated calls to arms of male employees.

It was against this backdrop that the pandemic spread along the country. From the analysed source, does evidence emerge confirming that pandemic can be modelled mainly as a dramatic and negative labour supply shock? In other words, did the overlapping of crises of a different nature (war, pandemic and social unrest) really prevent the flu, although extremely lethal and contagious, from causing a shock to the job offer, as predicted by economic theory?

The first reference to the Spanish Flu is found in a circular dated 14 September 1918, that is, at the very beginning of the second wave that would have been the deadliest of all:

*We understand that in various places a form of infectious fevers has manifested itself which, although not constituting any danger, spreads easily. Given the nature of the infection, we believe it is beneficial to draw the attention of the management of our branches to the opportunity that each one takes the most suitable measures to prevent, as far as possible, its own personnel from the spread of such fevers. We leave it to your initiative to give, after hearing the opinion of your doctor, or in correspondence with local health conditions, all those provisions or apply those measures that you consider most suitable for the special circumstances. We therefore believe it appropriate to point out that against the spread of this disease the best preventive defence consists in an effective intensification of normal hygiene measures, with abundant and frequent periodic disinfection of the premises*.^15^

The circular still flaunts certain tranquillity (“*although not constituting any danger*”), as it underestimates the flu spreading that was only then beginning to manifest itself with all its virulence.

In the short space of a week, however, the perception of events had already changed severely. A circular addressed to the branches on 21 September began to express deep concern about the possibility of guaranteeing the regular conduct of banking work, due to the spread of the flu among personnel already made scarce by calls to arms (it was the last stage of the conflict, with the maximum effort of men to be employed at the front):

*In view of the continuation of this [pandemic], we believe it useful to entertain you again on the subject to make you aware of the need to intensify the measures that you will no doubt have already taken and to require your staff to scrupulously comply with the general hygiene rules that were established by the local health authority, and the particular ones that your doctor were to advise*. […]

The countermeasures were no longer just those of an accurate disinfection of the premises, but real isolation began to be prescribed:

*It has now been proven that the spread of the disease occurs above all through contact with the sick, a contact that also depends on visits between colleagues and friends at the home of the affected; it will be necessary that you advise your staff to abstain absolutely from such visits. It will also be necessary to avoid contact of convalescent employees with other staff; you will therefore want to prescribe that they do not return to the bank without the explicit consent of your doctor; you will want to give instructions because, for the sick or suspected of an infectious form, adequate days of leave are prescribed so that the convalescence is in full out of the bank, and give a few more days of leave as a precaution.*

A sort of ‘green pass’ was also prescribed to return to work:

*Before resuming service, the recovered sick must undergo a new visit to the doctor, who, if any risk of contagion has disappeared, must explicitly declare whether the employee can resume service on the same day.*

So, unlike the earlier circular, the tone of this mail expressed the utmost concern, which was certainly destined to worsen shortly thereafter, when the infection reached its peak:

*We draw your attention to the great importance of this matter. The spread of the infection among our staff, already rarefied by calls to arms, could cause significant disturbance in our services; we therefore recommend that you apply the aforementioned general rules, and those measures that the special local circumstances make you deem most suitable with extreme promptness and energy, neglecting nothing to fight the infection and avoid its spread*.^16^

In October, the level of alarm was raised to the maximum, so much so that the communications of the central management to the branches took on a peremptory character as regards the only measure then available to combat the spread of the contagion, namely that of the isolation of sick people:

*Please make sure that employees who have suffered from the disease and are convalescing from the same, cannot return, even temporarily or incidentally, to the Bank’s premises if they have not undergone a new visit by your doctor, and if he has not declared in his report that they can immediately resume their service without risk of contagion for office colleagues*.^17^

At the end of October, the issue was no longer just an organizational one but also one of social stability. In fact, the increase in prices and the rage of the pandemic were increasing the distress of the staff, which the bank tried to cope with by granting one-off salary supplements equal to one/two months depending on the case.^18^

Since then, silence fell over the pandemic. Only on 20 January 1920 did a circular revealed the reappearance, in some centres, of “*not well-defined pandemic forms, which tend to spread also due to the unfavourable conditions of the season*”. It therefore referred to the prudential measures already sent to the branches between September and October 1918 “*to prevent poor general health conditions from affecting the efficiency of our staff*”.^19^ It was probably the Spanish Flu’s backlash.

If we look back over the circulars issued between October 1918 and January 1920, the reasons for this sudden disappearance of any concern about the epidemiological trend, at least as regards the risk of lack of personnel, emerge. After the autumn of 1918, the force of diffusion of the influenza had gradually been fading. Thus, the apex of the emergency lasted only a couple of months. Still, in light of the numbers of deaths and infections, one might expect a significant impact on the regular conduct of banking work in our reference institution.

However, the last months of 1918 were also the last of the war: on 24 October the Italian army began the offensive which led, on 4 November, to the defeat of the Austrian army and the signing of the armistice. The pandemic, therefore, soon gave way to other issues that circulars dealt with in the following months. These can be summarized as follows, with different effects on labour demand and supply.

The first issue concerned the return of Italian refugees to their lands of origin, which could have entailed a reduction in the number of available personnel, consisting precisely of emigrants who in the meantime had also been hired by Credito Italiano. In fact, hundreds of thousands of Venetians, Friulians, Trentino, Julians and Dalmatians had been forced to abandon their homes after the Italian rout of Caporetto in 1917. About 632,000 refugees fled throughout Italy from the various Venetian provinces during wartime. In 1918, however, the war ended and most of the refugees returned home (Riosa 2009, p. 247–248).

Credito Italiano was also significantly affected by such a potential labour reduction, since it involved

*a fairly significant amount of staff, who have been working for some time in our offices, and who, having become familiar with the work of the bank, could, in large part, find it convenient to remain employed by us if we were able to offer to them an occupation in the places to which they are about to return*.^20^

This possibility, therefore, strictly depended on the contextual territorial expansion that Credito Italiano, like other banking institutions, had planned to implement in the first post-war period. The projected opening of numerous branches, however, subjected the institute to further pressure on the front of finding additional staff.^21^

The second issue is related to the process of demobilization of army. Indeed, in front of events that reduced labour supply (pandemic and return of refugees) or increased demand (opening of new branches), other events pushed in the opposite direction instead. One of these was the need to reabsorb an increasing number of disabled ex-servicemen into the job market.^22^ Besides, the demobilization of the army represented a large pool of manpower to draw upon. The Credito Italiano’s central management was now giving instructions to the branches to rehire as many ex-employees as possible, who had had to resign because of the enlistment: the demobilization was rapid, so much so that, already in February of 1919, it was stated that “*the now advanced demobilization and the numerous exemptions granted have returned a large part of their employees called to arms to our branches*”.^23^

A third variable contributed to decreasing the labour demand, which overlapped the pandemic and the war, represented by the post-war social unrest (Fabbri 2009; Zamagni 1991). Social turmoil too had a major impact on labour organization, without a clear direct link to the Spanish Flu. The first evidence dates to May 1919, when the Credito Italiano’s Central Committee reported the request made in April by the Federation of Banking Personnel to obtain economic and regulatory improvements. Under the threat of a strike, the major Italian banks conceded an increase of the minimum wages and a reduction in working hours.^24^ Other personnel unrest was reported in May 1920: though severely punished by banks with disciplinary measures, strikes succeeded in obtaining further improvements in the contractual conditions. The upward wage push thus provided a strong incentive for Credito Italiano to make every effort “*to obtain maximum returns from existing personnel and avoid new hires that do not correspond to real and well-established service needs*”.^25^

## Discussion and conclusions

In this paper, we adopt an archival approach to evaluate the impact of the Spanish Flu on the labour market in Italy. In particular, we focus on the instructions issued between the end of 1914 and the middle of 1920 from the central management of Credito Italiano to local branches in order to cope with personnel management during the war and the pandemic period.

Archival sources offer some interpretative clues worthy of further study. Indeed, in the case of Credito Italiano (representative of a large banking company with a national dimension), our documentation shows that the Spanish Flu pandemic probably had little effect on the organization of work because the second wave – the deadliest one – lasted only a few months. Besides, despite the extraordinary number of global deaths, most influenza cases in 1918 were mild and indistinguishable from present-day influenza cases (Taubenberger and Morens 2006). Furthermore, the demobilization of the army was already underway throughout the third wave (the Vittorio Veneto battle ended in November, sanctioning the Italian victory in the war). It is not surprising, therefore, that the circulars sent to the branches at the end of the war dealt with issues other than the epidemiological condition (i.e. refugees, demobilization, branches opening, competition to hire more and more qualified personnel, also due to strikes and more expensive employment contracts).

In short, the Great War had a much stronger and structural impact, both during its development and in the subsequent period. Moreover, by inducing a change in the workforce composition (for instance, by stimulating the participation of women into the labour market to substitute men that were called to arms), the war made the system more resilient toward exogenous shocks.

Our results are in line with other papers adopting a quantitative methodology. For instance, Barro and Ursúa (2008), by running an econometric analysis about the impact on GDP of several historical events, argue that the impact of the Spanish Flu has been negligible if compared with that of other events as the World War I, the World War II, and the Great Depression. Karlsson et al. (2014), focusing on Sweden, find instead some effects of the Spanish Flu on the labour supply (but not on wages): notably, Sweden has not been directly involved in the World War I, thus suggesting that the (limited) evidence of the Spanish Flu impact on the labour market is probably due to the absence of the World War I as a confounding factor.

Anyway, a by-product of the pandemic event in Italy was that the banking institution became more attentive to the health conditions of its staff. The Spanish Flu’s experience increased the bank’s awareness of the permanent link between health risk *lato sensu* and economic risk, and that a similar tragedy could also come again. In this regard, the considerations expressed at the height of the pandemic on the role of the bank’s doctors appear emblematic:

*It is our desire that the task of the trusted doctors of our branches is not limited – in the visits made to personnel absent from the service due to illness – to ascertain the nature and probable duration of the illness, but whenever it is the case, extends to the investigation of the hygienic conditions in which the patient finds himself and the possibility to meet the needs of treatment. We are drawn to this from the intention of bringing help to the neediest personnel in the painful circumstances of infirmities, as well as from the desire to minimize the duration of absences due to illness, which are so damaging to the regular performance of services and accounting work*.^26^

Similarly, the instructions given to branches regarding the advisability of vaccinating staff are illuminating:

*Of course, vaccination is not compulsory: however, it is necessary to persuade all members of the staff to agree to undergo vaccination*.^27^

The same changed conditions of the labour market in the aftermath of the war (higher labour costs borne by companies) imposed

*to make health checks more rigorous at the time of recruitment, to avoid the entry into service of elements that from a physical point of view do not give full guarantee of assiduity at work and also to decrease the probability of having to cause dissolution of contract for health reasons, measures that are particularly unfortunate and burdensome*.^28^

Overall, our archival analysis highlights the importance to put the predictions of general economic models into a complex interpretative framework where historical and sociological aspects are properly taken into account. In particular, the archival analysis we propose in this paper could be used to complement other analytical traditions – quantitative and/or purely theoretical approaches. When quantitative methods are difficult to use due to the lack of reliable date (as in the case of old historical events) and theoretical models might find it difficult to stylize complex events (as a pandemic) involving sociological and historical aspects, archival analysis offers an alternative route to get insights. It also makes a compelling case for addressing economic and health shocks by combining diverse sources and methodologies.

Needless to say, the case study does not allow for generalization, suggesting reproducing this methodology also for other cases and countries, differentiating from a geographical and sectoral point of view. At this stage of the research, the archival evidence illustrated in Sect. 3 points out that the Spanish Flu may not have been sufficient to generate a negative shock on the labour supply in Italy due to the concomitant occurrence of other events which largely dominated the impact of the Spanish flu both in the organization of labour and in the perception of the contemporaries.

## Footnotes

1 For instance, the dispute about of the impact of the Black Death pandemic – by large the most devastating pandemic in the human history – is mainly due to the difficulty of having uncontroversial data (see for instance Munro, 2009).

2 See Berbenni and Colombo (2021) for a comprehensive discussion of the main macroeconomic models which are used to model pandemics.

3 Such a negative impact of the pandemic on the growth rate of the economy in the absence of saving and fertility variations could be basically imputed to the decreasing returns of capital accumulation. See Boucekkine et al. (2008) for details.

4 Following the tradition in pandemic modelling, this paper mainly focuses on the consequences of pandemic on the supply of labour. However, with regard the recent COVID-19 pandemic, some authors consider its implications on the demand side. For example, it is argued that the current pandemic has induced a radical change in the labour routines (Bieńkowska et al. 2022). Home working has become common and it is gradually replacing in-office working. This is likely to modify the skills that workers are expected to have (for example the ability to interact remotely is becoming more and more important). From a more aggregate perspective, the COVID-19 pandemic has determined the growth of some industries (pharmaceuticals, online retailing…) while it downsized the importance of other sectors (travels, entertainment…), thus restructuring accordingly the demand of labour (see De Vet et al., 2021). While these micro and macro implications are likely to be relevant for the COVID-19 pandemic, at the best of our knowledge the literature has not emphasized similar impacts for previous pandemics (including the Spanish Flu).

5 There are other papers considering the implications of the pandemic on the GDP growth. For example, Brainerd and Siegler (2003) estimate that the growth rate in the ‘20s has been greater in those US states that have been mainly affected by the pandemic flu, thus suggesting a positive relation between the flu and the medium-term growth rate of the economy. In contrast, Bloom and Mahal (1997), by focusing on India, do not find any significant relationship between the Spanish Flu and production loss. Carillo and Jappelli (2022), by performing an econometric exercise on Italian data, find that Spanish Flu caused a decline in the per-capita GDP growth of about 6.5% which lasts for three years. This conclusion is in line with the aggregate impact estimated by Barro et al. (2020).

6 On the confounding effects of war, see for instance Glick and Taylor (2009).

7 Other papers – less close to the present one – evaluate the implications of the Spanish Flu in terms of its impact on human capital accumulation. For instance, Almond (2006), building on Black et al. (2007) theory of in-utero exposure, argues that the Spanish Flu damaged fetal health, thus lowering human capital accumulation in the subsequent cohorts of US citizens. A similar finding is reported by Nelson (2010), Lin and Lin (2014) and Percoco (2016), by focusing on Brazil, Taiwan, and Italy, respectively.

8 Our paper is somehow related also to a different strand of literature that investigates the impact of *wars* on economic variables (see for instance Barro, 1991, Barro and Lee, 1993, and Jong-A-Pin, 2009). However, there are several differences between the expected implications of a war and those of a pandemic. Conceptually, the principal impact of a war can be identified in terms of loss of *both* human lives and physical capital (Thies and Baum 2020), whereas the impact of a pandemic is driven by the loss of human lives, and not of physical capital. Not surprisingly, economic history has documented different effects of wars and pandemics. For instance, in an interesting paper by Jordà et al. (2020), the authors estimated various impacts of wars and pandemics throughout the centuries with regard to several indicators, including: real wages, real per-capita GDP, and real natural rate (see Jordà et al., 2020, pp.9–14), thus confirming the different nature of wars and pandemics.

9 See Erkoreka (2010) for a detailed analysis of the pattern of the Spanish Flu.

10 ASU-Credito Italiano-Direzione Centrale-Personale-Fascicoli Generali-34/1 Istruzioni alle Filiali, fasc. 1, 4 December 1914.

11 Ibid, 11 August 1915.

12 Ibid, 11 August 1915.

13 Ibid, 09 June 1916. Another reference to the issue of women’s employment is in Ibid, 13 May 1918.

14 Ibid, 09 June 1916.

15 Ibid, September 14, 1918.

16 Ibid, 21 September 1918.

17 Ibid, 4 October 1918.

18 Ibid, 31 October 1918.

19 Ibid, fasc. 3, 20 January 1920.

20 Ibid, fasc. 1, November 14, 1918; see also Ibid, 4 November 1918.

21 Ibid, 9 November 1918. Traces of this active recruitment policy are still found in 1920, when the fierce competition and difficulties in finding qualified personnel were complained of due to the higher salaries offered by some commercial or industrial companies (Ibid, fasc. 3, 5 and 22 January 1920).

22 Ibid, 26 October 1918.

23 Ibid, fasc. 2, 12 February 1919.

24 ASU-Credito Italiano, Verbali del Comitato centrale, 2 May 1919.

25 ASU-Credito Italiano-Direzione Centrale-Personale-Fascicoli Generali-34/1 Istruzioni alle Filiali, fasc. 3, 8 May 1920 and 18 June 1920.

26 Ibid, fasc. 2, 13 March 1919.

27 Ibid, 14 April 1919.

28 Ibid, 30 May 1919.
